# A Versatile Microparticle-Based Immunoaggregation Assay for Macromolecular Biomarker Detection and Quantification

**DOI:** 10.1371/journal.pone.0115046

**Published:** 2015-02-06

**Authors:** Haiyan Wu, Yu Han, Xi Yang, George G. Chase, Qiong Tang, Chen-Jung Lee, Bin Cao, Jiang Zhe, Gang Cheng

**Affiliations:** 1 Department of Chemical and Biomolecular Engineering, University of Akron, Akron, Ohio 44325, United States of America; 2 Department of Mechanical Engineering, University of Akron, Akron, Ohio 44325, United States of America; Texas A&M University, UNITED STATES

## Abstract

The rapid, sensitive and low-cost detection of macromolecular biomarkers is critical in clinical diagnostics, environmental monitoring, research, etc. Conventional assay methods usually require bulky, expensive and designated instruments and relative long assay time. For hospitals and laboratories that lack immediate access to analytical instruments, fast and low-cost assay methods for the detection of macromolecular biomarkers are urgently needed. In this work, we developed a versatile microparticle (MP)-based immunoaggregation method for the detection and quantification of macromolecular biomarkers. Antibodies (Abs) were firstly conjugated to MP through streptavidin-biotin interaction; the addition of macromolecular biomarkers caused the aggregation of Ab-MPs, which were subsequently detected by an optical microscope or optical particle sizer. The invisible nanometer-scale macromolecular biomarkers caused detectable change of micrometer-scale particle size distributions. Goat anti-rabbit immunoglobulin and human ferritin were used as model biomarkers to demonstrate MP-based immunoaggregation assay in PBS and 10% FBS to mimic real biomarker assay in the complex medium. It was found that both the number ratio and the volume ratio of Ab-MP aggregates caused by biomarker to all particles were directly correlated to the biomarker concentration. In addition, we found that the detection range could be tuned by adjusting the Ab-MP concentration. We envision that this novel MP-based immunoaggregation assay can be combined with multiple detection methods to detect and quantify macromolecular biomarkers at the nanogram per milliliter level.

## Introduction

The quantitative detection of biomarker(s) is very important in clinical diagnostics [1, 2] environmental monitoring [3, 4] and a variety of other biological research [5]. Among various types of biomarkers, macromolecular biomarkers, such as antibodies, glycoproteins and enzymes, have recently attracted increased interest due to their presence in various diseases [6, 7]. To detect macromolecular biomarker, immunoassay is a prevalent method due to its high specificity. However, conventional immunoassays, such as enzyme-linked immunosorbent assay (ELISA) [8], surface plasmon resonance (SPR) [9, 10], and quartz crystal microbalance (QCM) [11] require relative long assay times, and typically employ bulky and complicated detection instruments. Additionally these methods require either enzyme or fluorescence labeling of antibodies [12] or the modifications of sensing surfaces [13]. A fast, highly sensitive and low cost immunoassay method, which does not require complex sample preparations or complex detection instrumentation, is urgently needed for laboratories and clinics lacking immediate access of analytical instruments [14]. Furthermore, this immunoassay method should be compatible with commonly used analytical lab instruments.

**Fig 1:**
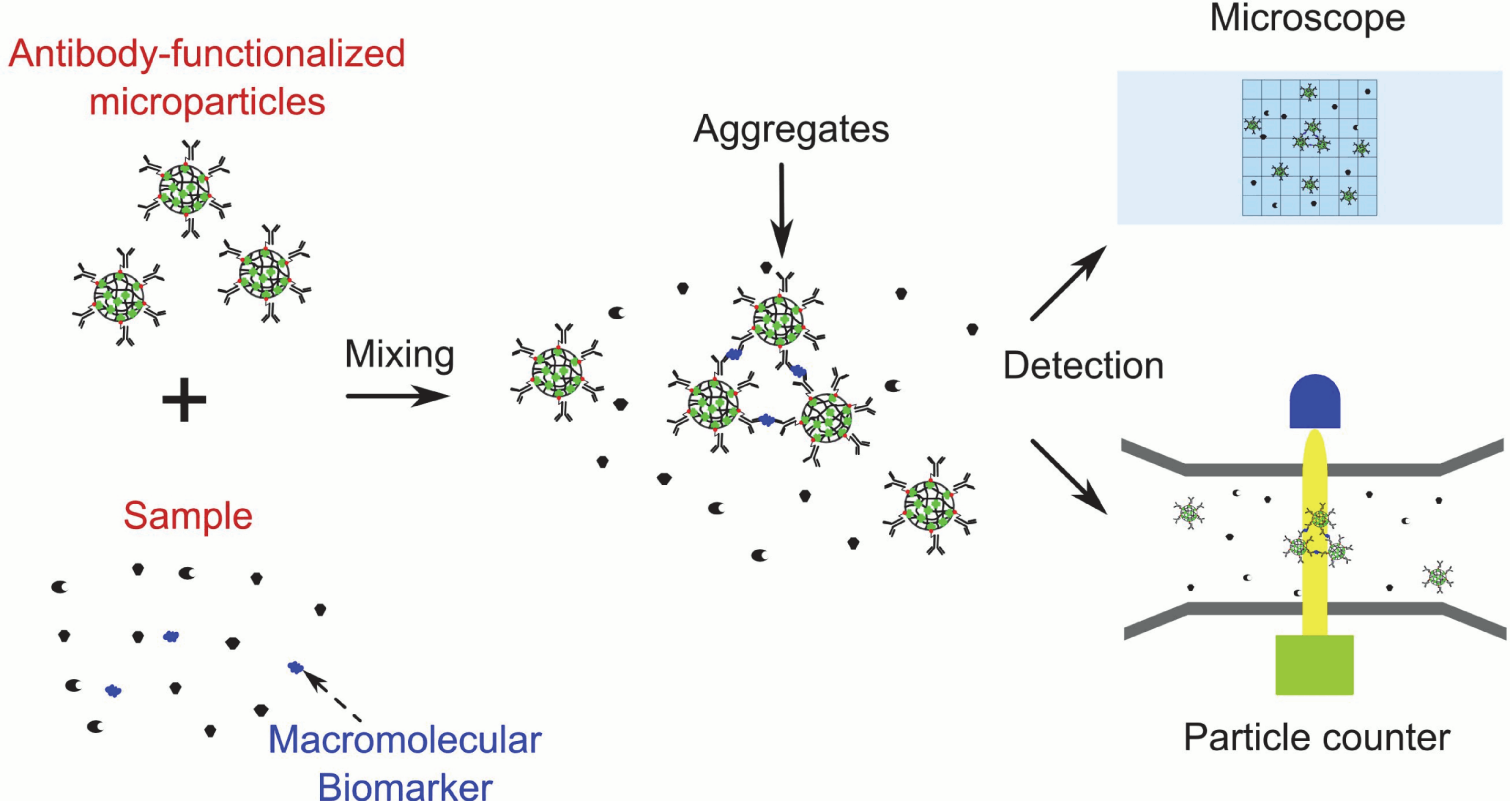
Illustration of the principle of immunoaggregation assay, which can be readily coupled with optical microscopes or particle counters for quantitative and qualitative detection of biomacromolecules.

The objective of this work was to develop a sensitive, low cost and versatile microparticle (MP)-based immunoaggregation assay, for the quantitative and qualitative detection of macromolecular biomarkers. Fig. 1 illustrates the concept of the simple and innovative MP-based immunoaggregation assay reported in this study. It was expected that the macromolecular biomarkers could cause the aggregation of antibody (Ab)-functionalized MPs. Ab-MP aggregates could be detected by either a simple optical microscope or the high throughput optical or electrical particle counting device. In this work, we developed the immunoaggregation assay protocol and demonstrated the concept of immunoaggregation using goat anti-rabbit IgG and human as two model biomarkers. Both the number fraction and the volume ratio of Ab-MP aggregates to all particles were clearly related to the concentration of the biomarker.

## Materials and Methods

Streptavidin–functionalized Microparticle (MP) (Dynabeads M-280 with a diameter of 2.8 μm), biotinylated polyclonal rabbit anti-goat IgG (rAb) and goat anti-rabbit IgG (goat IgG) (labeled with Alexa Fluor 488) were bought from Life Technologies (Carlsbad, CA, USA). Goat anti-human ferritin polyclonal antibody (gAb) and human ferritin were purchased from United States Biological (Salem, MA, USA). NHS-Fluorescein, NHS-PEG4-Biotinyltion and Zeba spin desalting column were purchased from Thermo Scientific (Waltham, MA, USA). Dimethyl sulfoxide (HPLC grade) was bought from Alfa Aesar (USA). Phosphate buffer saline (PBS, pH 7.4), and bovine serum albumin (BSA) were obtained from Sigma-Aldrich (St Louis, MO, USA).

To prepare the immunoaggregation sample, MP and biotinylated rAb were diluted to 0.16 mg/mL and 6.4 ng/mL separately in PBS containing 0.1% BSA. Equal volumes of 166.7 μL of diluted MP solution and 166.7 μL of diluted rAb solution were mixed for 30 minutes on a thermo mixer agitated at 650 rpm at room temperature. Biotinylated rabbit anti-goat Abs were conjugated to MP to form rAb-MP through the streptavidin-biotin binding. The conjugated solution was placed on a magnet to separate rAb-MPs from the solution and the unconjugated Ab supernatant was discarded. The rAb-MPs were resuspended with PBS with 0.1% BSA to the concentrations of 53.4 μg/mL. Different concentrations of goat IgG, which was used as model biomarker, were prepared with a range from 0.1 ng/mL to 320 ng/mL. 333.4 μL of Ab-MP solution was mixed with 166.7 μL of goat IgG solutions at different concentrations for 30 min on a thermal mixer at 650 rpm at room temperature. The same procedure was used for human ferritin detection. Goat anti-human ferritin polyclonal antibody (gAb) functionalized MP (gAb-MP) were suspended in PBS with 0.1% BSA to two different concentrations, 53.40 μg/mL and 213.4 μg/mL. The concentration of human ferritin ranged from 0.1 ng/mL to 416 ng/mL in PBS with 0.1% BSA. In a parallel study, 10% fetal bovine serum (FBS, Sigma-Aldrich, USA) was used to dilute the human ferritin to different concentrations ranging from 0.1 ng/mL to 416 ng/mL to mimic the biomarker detection in the complex medium.

To characterize the immunoaggregation, rAb-MP solutions with and without biomarker (goat IgG) were imaged with a fluorescent microscope (IX-81, Olympus, Japan) under a 40X objective lens though bright field filter and GFP filter (494/518 nm) respectively and analyzed with MetaMorph microscopy automation & image analysis software (Molecular Devices, CA, USA). The rAb-MP aggregates solutions formed under different goat IgG concentrations were dropped to a glass slide and covered with a cover slip. The number of rAb-MP, 2-rAb-MP aggregates, 3-rAb-MP aggregates and 4-rAb-MP aggregates were counted separately through the Olympus IX-81 fluorescent microscope under the bright field mode. To ensure accuracy and repeatability, more than 1000 particles were counted for each sample and 3 samples were prepared and tested for each goat IgG concentration. The number fraction of aggregates (*fn*) was defined as the ratio of the number of individual particles in aggregates to the number of all individual particles. The same characterization methods were used for the human ferritin detection. To confirm the result, Ab-MP aggregates were diluted to 100 mL PBS with 0.1 mg/mL BSA and the size and counts of the particles in the sample were measured using a particle counter with a detection range of 0.5∼500 μm (Accusizer 780, PALS-Particle Sizing Systems, FL, USA). The volume fraction of aggregates (*fv*) was calculated as volume ratio of large particles (3.0 μm∼10 μm) to all particles (1.5 μm ∼10 μm).

## Results and Discussion

**Fig 2:**
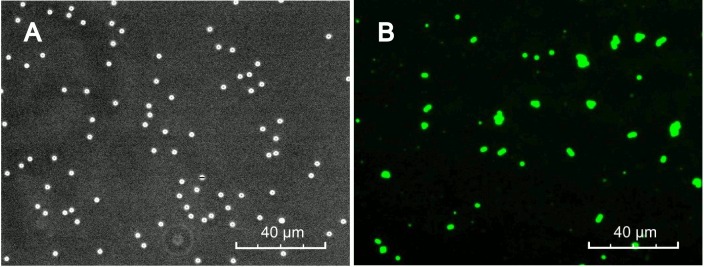
Fluorescence Microscope images: (A) FITC labeled rAb-MP without goat Ig G and (B) FITC labeled rAb-MP with 36 ng/mL goat IgG as a model biomarker. The concentration of rAb-MP was kept constant at 53.4 μg/mL.

**Fig 3:**
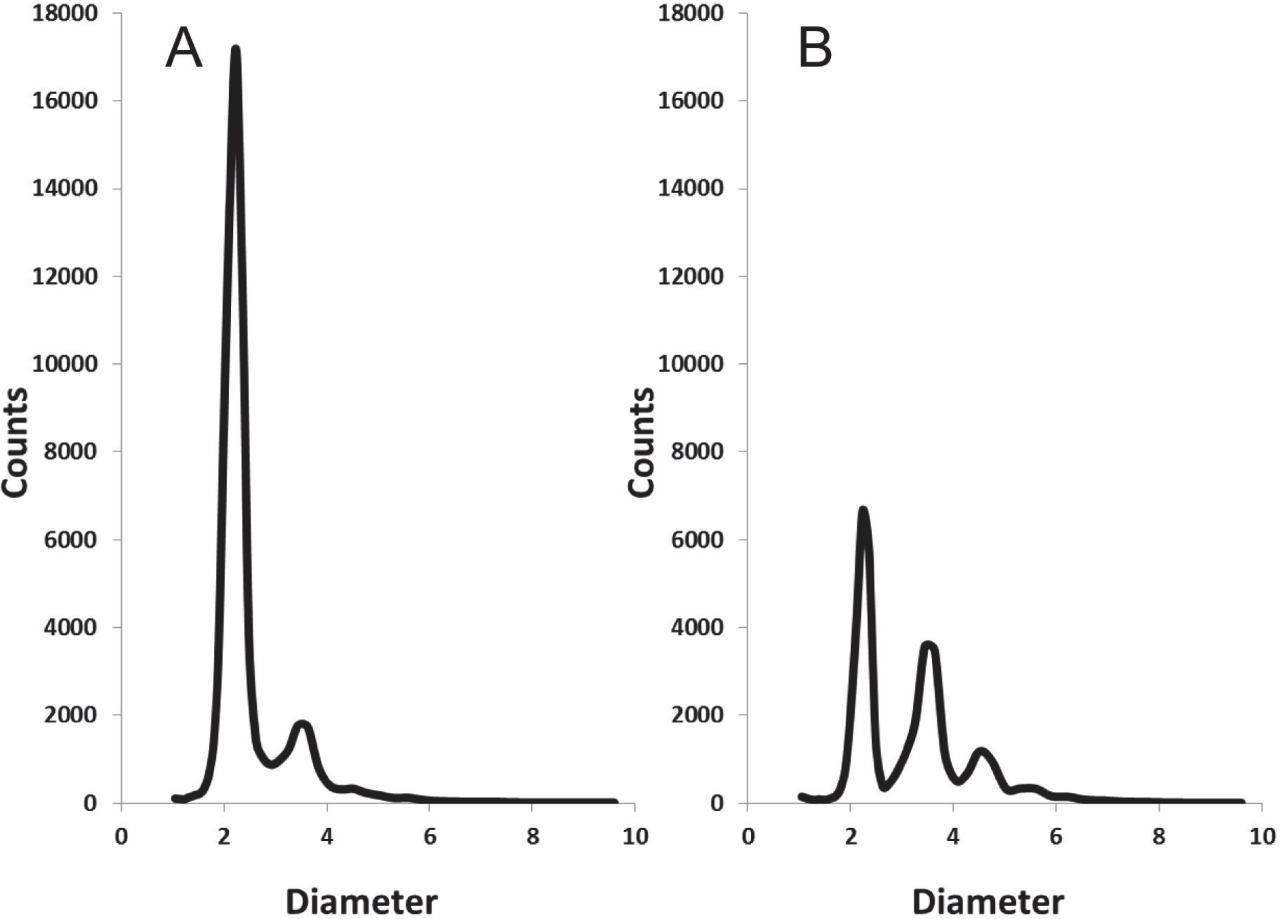
Accusizer measurement results for (A) FITC labeled rAb-MP without goat Ig G and (B) FITC labeled rAb-MP with 36 ng/mL goat IgG as a model macromolecular biomarker. The concentration of rAb-MP was kept constant at 53.4 μg/mL.

To prove the concept of immunoaggregation, goat IgG was used as a model biomarker and FITC-labeled rAb was conjugated to MP as a capture probe. The qualitative aggregation phenomenon of rAb-MP was studied using the fluorescent microscope though a GFP filter. Fluorescent microscope images (Fig. 2) show the dispersion state of particles in the rAb-MP solution of 53.4 μg/mL and the rAb-MP solution mixed with goat IgG with a final concentration was 36 ng/mL. In the absence of goat IgG, most rAb-MPs uniformly distributed and mostly separated from each other. After mixed with goat IgG, a large amount of rAb-MP aggregates formed. Fig. 3 shows the particle size distribution for rAb-MP solution with and without goat IgG at the same concentration with previous microscope study, which were measured by Accusizer. In both samples, multiple peaks were observed in the particle size frequency distribution curves generated by the Accusizer software, however individual particles (< 3 μm) dominated (89%) in MP and Ab-MP samples. The small amount of larger particles (>3 μm) were formed by the nonspecific aggregation of individual particles. As shown in Fig. 3B, the percentage of larger particles ranging from 3 μm to 6 μm dramatically increased, which indicated the rAb-MP aggregates went up. The decrease of individual particles was caused by the aggregation triggered by the antigen. The result indicates that a large amount of rAb-MP aggregates can be formed because of the addition of biomarker.

**Fig 4:**
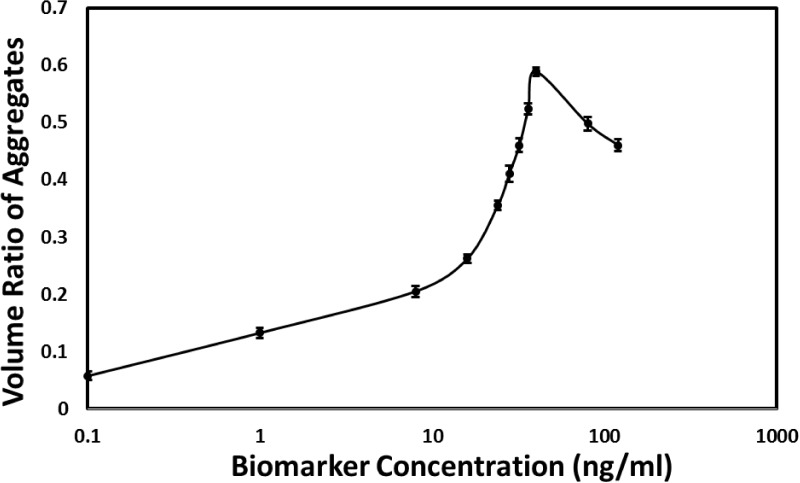
The number fraction of rAb-MP aggregates to all particles as a function of goat IgG concentration in PBS containing 0.1% BSA. Particle counts were obtained from bright field microscope images. The standard deviation was calculated from three replicates.

Quantitative detection of biomarker is needed for many applications. We hypothesized that at the given particle concentration, the number or volume ratio of aggregates to total particles is proportional to the antigen concentration. To test our hypothesis, the immunoaggregation behaviour of rAb-MP as a function of goat IgG concentration in PBS solution was measured by using the IX-81 microscope under the bright field mode. The concentration of rAb-MP was kept constant at 53.4 μg/mL and mixed with different concentrations of goat IgG ranging from 0.1 ng/mL to 320 ng/mL. Single rAb-MP and 2-rAb-MP aggregates, 3-rAb-MP aggregates and 4-rAb-MP aggregates for each goat IgG concentrations were recognized and counted separately through recorded microscope images. The aggregates formed by more than 4 rAb-MPs were neglected in the calculation, since they were less than 1%. To evaluate the nonspecific aggregates, the negative control sample of rAb-MP in PBS without goat IgG was also counted; the number fraction of nonspecific rAb-MP aggregates was 6.8 ± 0.01%. The number fraction of rAb-MP aggregates for each goat IgG concentration was calculated by subtracting the nonspecific aggregates volume fraction value (6.8%) and had a clear relationship with goat IgG concentration as shown in Fig. 4. At least 3 samples were prepared and counted for each goat IgG concentration. The max number fraction (*fn* = 65.7%) of rAb-MP aggregates were achieved at 40 ng/mL of goat IgG. Within the range from 0.1 ng/mL to 40 ng/mL, the volume fraction of aggregates went up with the increase of goat IgG concentration, while it went down with the increase of goat IgG concentration above the turning point (40 ng/mL). This is because goat IgG at high concentrations saturated rAb on MPs; therefore the number of unreacted rAb on MP was too low to aggregate.

**Fig 5:**
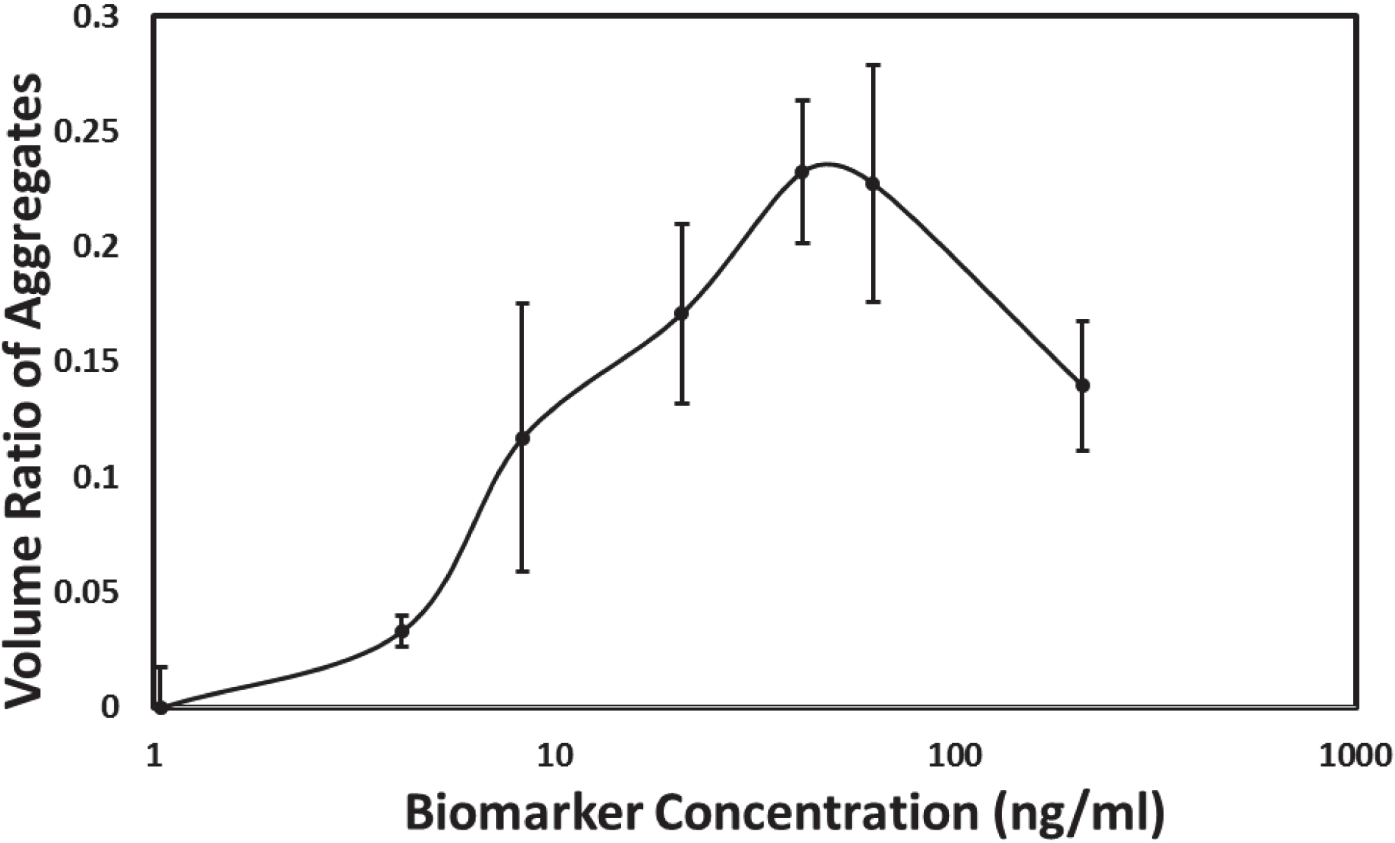
The volume fraction of gAb-MP aggregates to all particles as a function of human ferritin concentration in PBS containing 0.1% BSA. Particle counts were obtained from Accusizer. The standard deviation was calculated from five replicates.

Human ferritin was used as a real biomarker to validate whether MP-based immunoaggregation assay could be applied for the real human biomarker detection. Ferritins exist in many organisms, including vertebrates, invertebrates, plants, fungi, and bacteria, and they function as iron storage proteins [15]. The abnormal level of ferritin in serum can be used as an indicator of various human disease, such as tumors and infectious microorganisms [16, 17] Ferritin measurement is considered to be a reliable method for the evaluation of iron stores [18]. Goat anti-human ferritin polyclonal antibody (gAb) were conjugated with MP to form gAb-MP and used as a capture probe. The samples with different concentrations of human ferritin antigen ranging from 1.04 ng/mL to 104 ng/mL in PBS and the constant gAb-MP concentration (53.4 μg/mL) were measured by the Accusizer. The volume fraction (*fv*) of gAb-MP aggregates (3.0 μm∼10 μm) to all particles (1.5 μm ∼10 μm) were calculated and were plotted versus ferritin concentration (Fig. 5). Since the accusizer does not directly provide the information about how many smaller particles a larger particle is composed of, the volume fraction (*fv*) of larger particles to all particles more directly reflects the aggregation behavior of gAb-MP in this case. To ensure repeatability, at each ferritin concentration, 5 samples were prepared and measured. Fig. 5 shows that the same trend with goat IgG as the biomarker: The maximum volume fraction (*fv* = 23.2%) of gAb-MP aggregates was achieved at the ferritin concentration of 41.6 ng/mL. Within the range from 1.04 ng/mL to 41.6 ng/mL, the volume fraction of gAb-MP aggregates to all the particles follows an upward trend with the increase of ferritin concentration. At higher ferritin concentrations (>41.6 ng/mL), the volume fraction of gAb-MP aggregates decreased with the increase of ferritin concentration.

**Fig 6:**
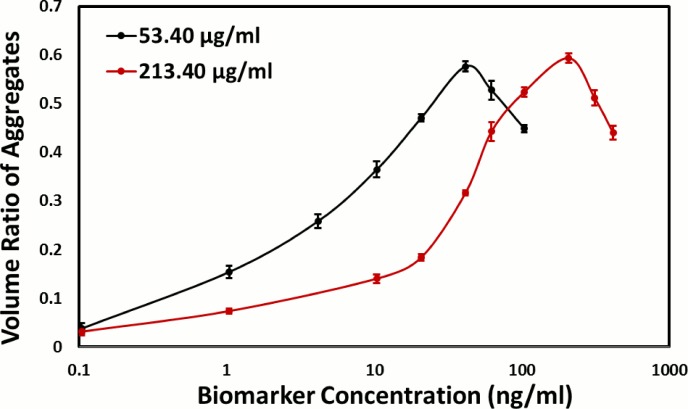
The number fraction of gAb-MP aggregates to all particles as a function of human ferritin concentration in 10% FBS at 53.40μg/mL (dish line) and 213.40μg/mL (solid line) of gAb-MP. Particle counts were obtained from bright field microscope images. The standard deviation was calculated from three replicates.

Detection of biomarkers is a challenge at low concentrations in complex media, such as body fluid, blood, urine, etc., since the nonspecific binding of biomolecules to the capture probes or sensing surfaces may cause the false positive result and decrease the detection sensitivity [19, 20]. To evaluate the feasibility of the immunoaggregation assay for biomarkers in a complex medium, we used 10% FBS as the solution to replace PBS with 0.1% BSA to mimic a real detection environment. gAb-MP at two final concentrations, 53.4 μg/mL and 213.4 μg/mL, were mixed with human ferritin solution in 10% FBS. The solution with 10% FBS and the same concentration of gAb-MP but without ferritin was used as the negative control. Each sample was observed using the microscope under bright field mode; individual gAb-MPs, 2-gAb-MP aggregates, 3-gAb-MP aggregates and 4-gAb-MP aggregates were counted separately. More than 1000 particles were counted for each sample and 3 samples were counted for each ferritin concentration. The number fraction (*fn*) of nonspecific aggregates of the negative control were 6.8 ± 0.3% and 6.8 ± 0.1% for gAb-MP concentrations of 53.4 μg/mL and 213.4 μg/mL respectively. The low number fractions of the nonspecific gAb-MP aggregation suggest that gAb-MPs were stable in 10% FBS. The number fraction of aggregates of each sample was obtained by subtracting the number fraction value of the nonspecific aggregates. Fig. 6 shows that for the lower gAb-MP concentration (53.4 μg/mL), the detection range was from 0.1 ng/mL to 62.4 ng/mL. The result demonstrates that microparticle-based immunoaggregation assay for biomarker detection is insensitive to other biomolecules in the complex media, implying that this method can be applied for biomarker detection in complex media.

Since different biomarkers exist at different concentrations, it is highly desired that the detection range of the assay be adjustable to match the various concentrations of different biomarkers. Based on the immunoaggregation principle, the detection range can be tuned by changing the Ab-MP concentration. Fig. 6. shows the detection range can be shifted to the higher concentration range (0.1 ng/mL to 208 ng/mL) by using a higher concentration of gAb-MP (213.4 μg/mL). Our study demonstrates that we can change the detection range by adjusting the Ab-MP concentration for different biomarkers. Furthermore, we can use two particle concentrations to validate the accuracy of the result for samples with an unknown concentration range.

We think this detection method will be particularly useful for hospitals or laboratories that need rapid clinical detection but lack immediate access to analytical instruments. Since the capture probe, Ab-MP conjugates could be prepared before the detection, the assay time for this method is less than 1 hour, which is shorter compared to the conventional ELISA method. Furthermore, the quantitative or qualitative detection of the biomarker can be conducted using optical microscopes with a hemocytometer, which are inexpensive and usually available in hospitals and biological labs. If we add equal amount of particles to different samples with the same volume, the concentration of total particles in all samples will be the same. Since the volume of the fluid in the hemocytometer is fixed, the number of the aggregates in the hemocytometer can be directly linked to the antigen concentration. Therefore, we only need to determine the number of aggregates instead of recording both the counts of aggregates and individual particles. Moreover, the software for counting the particles and aggregates, such as Image J, could be used to replace manual counting to further reduce the assay time. It is expected that complex samples, such as blood and body fluid, may cause higher non-specific aggregations that will increase the noise and detection limit. However, the detection limit of for our method is 0.1 ng/mL, which is lower than that of commercial available human ferritin ELISA kits (approximately 1 ng/mL), so the blood sample can be conveniently diluted to a suitable concentration before detection. On the other hand, as we demonstrated, the detection range can be tuned by adjusting the Ab-MP concentration. For concentrated blood sample, a lower Ab-MP concentration can be used to achieve a lower detection range. The same method can be used to detect multiple biomarkers simultaneously. Microparticles with different color and capture probes can be premixed and then added to the sample. Each type of the biomarker will cause the aggregation of microparticles with the specific color. If the sample contains different types of biomarkers, aggregates with different color can be detected. For the rapid qualitative detection, given the fixed sample volume and particle concentration, the threshold value of aggregates can be predetermined and sample can be considered as the positive once the number of aggregates is over the threshold value.

## Conclusions

An innovative MP-based immunoaggregation assay for macromolecular biomarker detection was demonstrated. The biomarker can cause the aggregation of Ab functionalized microparticles and the number or volume fraction of aggregates increased with the concentration of biomarker among the detection range. The detection range is 0.1 ng/mL to 40 ng/mL and 0.1 ng/mL to 208 ng/mL for two model biomarkers, goat IgG and human ferritin, respectively. The detection range can be further extended by adjusting the Ab-MP concentration. It has also been proved that the MP-based immunoaggregation assay can be applied to real biomarker in 10% FBS, which is close to the real detection environment. To the best of our knowledge, it is for the first time that the immunoaggregation method was reported for biomarker detection. This novel MP-based aggregation immunoassay could potentially be used to detect macromolecular biomarkers at the ultra-low concentration (nanogram per milliliter) without using expensive and dedicated analytical instruments.
